# Multiplex enhancer-reporter assays uncover unsophisticated TP53 enhancer logic

**DOI:** 10.1101/gr.204149.116

**Published:** 2016-07

**Authors:** Annelien Verfaillie, Dmitry Svetlichnyy, Hana Imrichova, Kristofer Davie, Mark Fiers, Zeynep Kalender Atak, Gert Hulselmans, Valerie Christiaens, Stein Aerts

**Affiliations:** 1 Laboratory of Computational Biology, Center for Human Genetics, University of Leuven, 3000 Leuven, Belgium;; 2 VIB Center for the Biology of Disease, 3000 Leuven, Belgium

## Abstract

Transcription factors regulate their target genes by binding to regulatory regions in the genome. Although the binding preferences of TP53 are known, it remains unclear what distinguishes functional enhancers from nonfunctional binding. In addition, the genome is scattered with recognition sequences that remain unoccupied. Using two complementary techniques of multiplex enhancer-reporter assays, we discovered that functional enhancers could be discriminated from nonfunctional binding events by the occurrence of a single TP53 canonical motif. By combining machine learning with a meta-analysis of TP53 ChIP-seq data sets, we identified a core set of more than 1000 responsive enhancers in the human genome. This TP53 *cistrome* is invariably used between cell types and experimental conditions, whereas differences among experiments can be attributed to indirect nonfunctional binding events. Our data suggest that TP53 enhancers represent a class of unsophisticated cell-autonomous enhancers containing a single TP53 binding site, distinct from complex developmental enhancers that integrate signals from multiple transcription factors.

Enhancers are essential regulatory elements that are bound by transcription factors (TFs) to shape the gene expression network underlying a cellular phenotype. Understanding the enhancer code is crucial to achieve a functional regulatory annotation of the human genome, which is ultimately required to understand developmental processes and disease-related variation in the noncoding part of the genome. However, the complexity of the enhancer logic, their sparse distribution, context-specificity, and distal location from genes in the genome make it challenging to identify and validate enhancers. During recent years, high-throughput sequencing efforts like ENCODE and the Epigenomics Roadmap have yielded an enormous wealth of regulatory data (Gerstein et al. 2012; Roadmap Epigenomics Consortium et al. 2015). Some of the most commonly used approaches are chromatin immunoprecipitation (ChIP-seq) to localize regions bound by a certain TF or a modified histone, and various assays aimed at localizing accessible and free regions within the genome (e.g., DNase I hypersensitive sites sequencing [DNase-seq] or Assay for Transposase-Accessible Chromatin using sequencing [ATAC-seq]). Although these methods generate genome-wide regulatory profiles and chromatin states (Ernst et al. 2011), they have not led to sequence-based enhancer models and usually contain high levels of nonfunctional noise.

A key feature of TF binding is the presence of the TF's DNA recognition sequence. Interestingly, sequence analysis on ChIP-seq data in yeast showed that not all identified peaks are directly bound by their TFs (Gordân et al. 2009). Similarly, among the hundreds of TF ChIP-seq data of ENCODE, only a fraction of peaks were found to contain the canonical recognition sequence of their respective TF (Wang et al. 2012). Although a considerable amount of ChIP-seq peaks seem to be independent of the TF motif, it is not clear whether this indirect binding plays a functional role, i.e., whether it is involved in the regulation of a target gene. In *Drosophila*, it has been suggested that indirect binding could be functional and contribute to gene regulation via transcription factor cooperative activity, i.e., the tethering of a TF to an enhancer by other, directly bound factors (Junion et al. 2012). On the other hand, indirect binding could also reflect a technical aspect of ChIP and represent for instance fixation artifacts (Waldminghaus and Skarstad 2010; Keren and Segal 2013; Baranello et al. 2015).

Using the motif of the bound factor may be a good guide to identify functional binding, but for most (if not all) TFs, tens of thousands of recognition sequences, present throughout the genome, remain unoccupied or unbound. The current paradigm of how transcription factors discriminate between functional and nonfunctional locations is based on the combinatorial action of transcription factors. Here, binding specificity is achieved through clusters of binding motifs co-occurring within an enhancer (Panne 2008; Maston et al. 2012; Lee and Young 2013; Shlyueva et al. 2014; Stampfel et al. 2015) or by generating new recognition sequences for pairs of TFs (Jolma et al. 2015). Such enhancers are generally classified under the enhanceosome or billboard models, depending on whether or not the order and spacing of motifs is important (Arnosti and Kulkarni 2005). Other local features in the DNA sequence of an enhancer have also been shown to contribute to the discrimination of bound versus unbound sites, such as GC content (White et al. 2013), preferential sequences for nucleosome positioning (Lidor Nili et al. 2010), DNA shape features (Chiu et al. 2015), and dinucleotide repeat motifs (Yáñez-Cuna et al. 2014). However, to gain further insight into the *cis*-regulatory enhancer code and identify those sites that are truly bound and functional, validation assays are needed.

The recent development of multiplex enhancer-reporter assays has been invaluable in procuring such information on a large scale. Whereas classically enhancer-reporter assays consist of cloning each enhancer one by one, first in vitro, later in vivo (Banerji et al. 1981; O'Kane and Gehring 1987; Chiocchetti et al. 1997; Dailey 2015), now hundreds to thousands of enhancers can be tested in parallel (Patwardhan et al. 2009, 2012; Kwasnieski et al. 2012; Melnikov et al. 2012; Arnold et al. 2013; Kheradpour et al. 2013; Smith et al. 2013; White et al. 2013; Vanhille et al. 2015). These methods can be broadly categorized in two groups, namely, massively parallel reporter assays (MPRA) utilizing barcodes as a measure of activity of synthesized enhancer fragments (Patwardhan et al. 2009, 2012; Kwasnieski et al. 2012; Melnikov et al. 2012; Kheradpour et al. 2013; Smith et al. 2013; White et al. 2013) and self-transcribing active regulatory region sequencing (STARR-seq) (Arnold et al. 2013; Vanhille et al. 2015).

In this work, we unravel the genome-wide binding profile of TP53 (protein product of *TP53*, also known as p53), addressing these two questions simultaneously by investigating (1) the differences between direct and indirect binding; and (2) the differences between bound and unbound recognition sequences in the genome. TP53 is a tumor-suppressor that regulates its target genes in response to different stimuli like DNA damage or cellular stress, eliciting functions like growth arrest or apoptosis (Vousden and Prives 2009). Its importance is moreover reflected by the fact that *TP53* is the most commonly mutated gene found in cancer (Muller and Vousden 2014). Although much research has gone in understanding how and where TP53 interacts with DNA, many questions and contradictions remain. For instance, it is unclear what the functional role is of indirect binding of TP53 to the DNA (Kirschner et al. 2015), whether TP53 also directly represses genes (Johnson et al. 2001; Ho and Benchimol 2003; Rinn and Huarte 2011), and how TP53 elicits different responses like apoptosis and growth arrest, activating different genes for each process (Smeenk et al. 2008; Menendez et al. 2013). Several elements of the TP53 binding site architecture have been proposed to contribute to the functional properties of the enhancer. These include variations of the spacer between two TP53 half-sites (Hoffman et al. 2002; Godar et al. 2008), or variations in binding sites for coregulatory factors (Koutsodontis et al. 2001; Thornborrow and Manfredi 2001). However, these properties have not been evaluated on a global scale. To resolve these uncertainties and to generate a global TP53 enhancer model, we have combined two types of multiplex enhancer-reporter assays followed by machine learning. Our results yield a new unsophisticated model for TP53-mediated transcriptional regulation and allow us to create a ranked list of all potentially functional TP53 binding sites in the human genome.

## Results

### Quantitative enhancer-reporter activity for hundreds of enhancers in parallel

**Figure 1.**
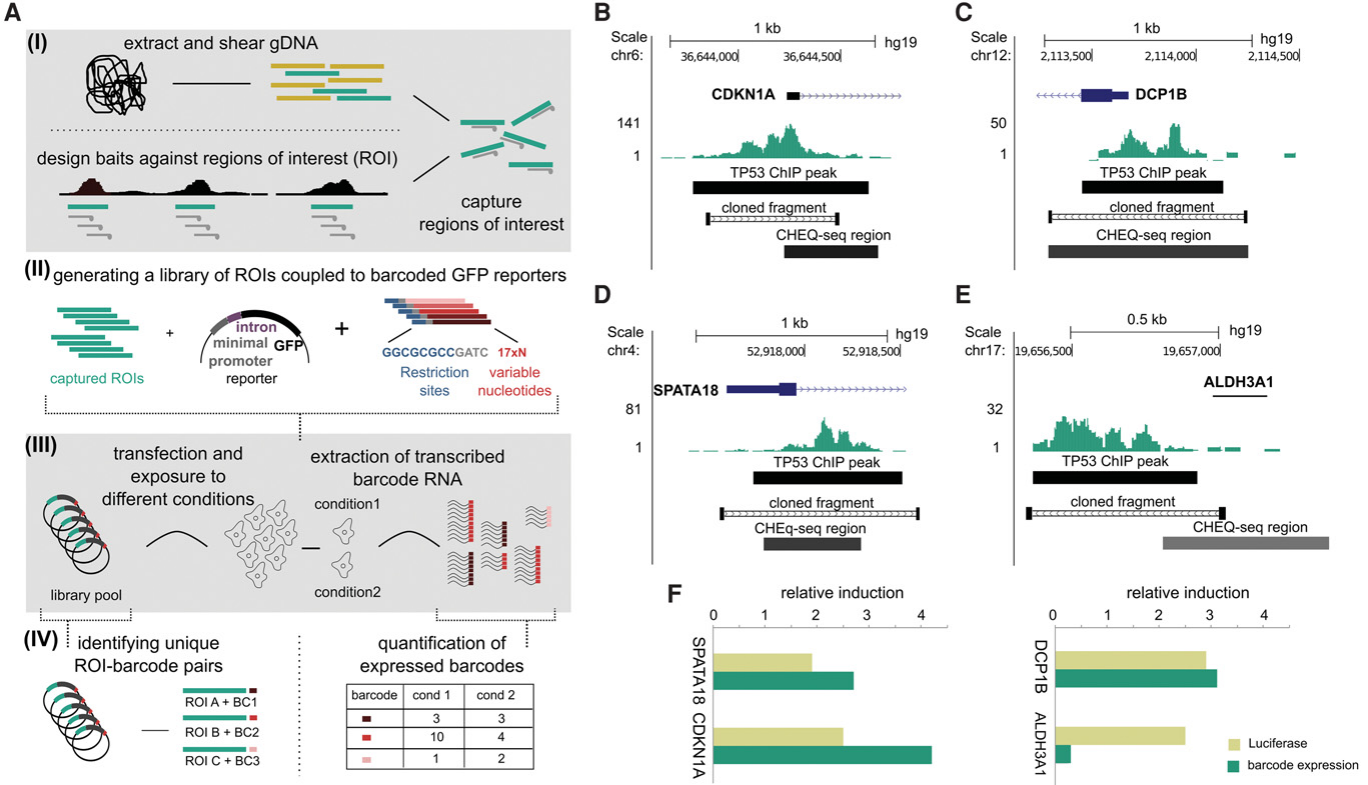
Overview of the CHEQ-seq reporter assay. (*A*) (I) Genomic DNA is sheared and custom baits are used to capture the regions of interest (ROI). (II) Captured ROIs are cloned into a reporter library consisting of a GFP-based reporter linked to a pool of 17 × 10^9^ barcodes. (III) The reporter library is transfected under various conditions, after which the RNA of transcribed barcodes is extracted. (IV) Randomly coupled ROI-barcode couples are identified using PacBio sequencing, and barcode expression is measured using Illumina short-read sequencing. (*B*–*E*) Four TP53 ChIP-seq peaks comparing the CHEQ-seq barcode level with luciferase activity of a manually cloned fragment. (*F*) CHEQ-seq and luciferase induction only agree when they both overlap with the ChIP-seq peak summit.

We developed a new method, called Captured High-throughput Enhancer testing by Quantitative sequencing (CHEQ-seq), to test enhancer-reporter activities for hundreds of enhancers simultaneously (Fig. 1A). Although conceptually CHEQ-seq is similar to the recently published capture-and-clone variant of CRE-seq (Kwasnieski et al. 2012; Shen et al. 2015), CHEQ-seq uses a different cloning and sequencing strategy (Supplemental Protocol). First, candidate enhancers from sheared genomic DNA are captured using custom designed baits (Fig. 1A; Supplemental Fig. S1A). These enriched DNA fragments, averaging 500 base pairs, are then cloned upstream of a fluorescent protein reporter preceded by a minimal promoter and a synthetic intron (Arnold et al. 2013) and followed by a 17 base pair random barcode allowing for 17 × 10^9^ possible barcodes (Methods). Using CHEQ-seq, we tested the enhancer activity of 1526 TP53 ChIP-seq peaks obtained in MCF7 breast cancer cells treated with Nutlin-3a (Janky et al. 2014). Additionally, 94 promoters of housekeeping genes (HKG) were selected as control regions, assuming that they drive stable reporter gene expression independent of any perturbation (Eisenberg and Levanon 2013). Also, 66 negative control regions in the genome were selected (Methods; Yip et al. 2012). Unique to CHEQ-seq is long-read sequencing of the entire library to resolve the randomly formed enhancer-barcode pairs, which after processing and removing redundancy, yielded 20,751 distinct genomic fragments linked to 24,906 different barcodes (Methods; Supplemental Figs. S1B, S2).

To test which candidate enhancers are TP53-responsive, we transfected the enhancer-reporter library into MCF7 cells treated with Nutlin-3a, activating TP53 (p53-high) or containing a stable shRNA knockdown for *TP53* (p53-off) (Supplemental Fig. S1A; Phillips et al. 2010). Reporter activity levels were determined by counting sequencing reads of barcoded cDNAs and were normalized both by the input library and by the re-extracted plasmid DNA (Methods; Supplemental Fig. S3). Of all the barcodes linked to a genomic fragment, we could measure 21,182 distinct barcodes representing 18,399 unique genomic fragments, of which 22.8% overlapped with the designed regions (81,428-fold enrichment) (Supplemental Table S1; Supplemental Fig. S1C).

We performed several quality control steps to assess whether the barcode expression represents accurate enhancer-reporter levels. First, independent biological replicates show a very high correlation among barcodes within the p53-high condition (*r*^2^ = 0.99). The correlation of the p53-off condition is much lower, as expected, since the majority of enhancers are inactive in the p53-off condition (*r*^2^ = 0.56). The induced activity, measured as the fold-change between p53-high and p53-off, also correlates between the two biological replicates (*r*^2^ = 0.72) (Supplemental Fig. S4). Second, the expression levels of two different barcodes linked to the same enhancer region showed strong correlation of the fold-changes between p53-high and p53-off (*r*^2^ = 0.73). Third, different regions that overlap with the same designed region also show strong correlation (*r*^2^ = 0.72) (Supplemental Fig. S5). Finally, the CHEQ-seq enhancer-reporter differential expression values are recapitulated when performing classical enhancer-luciferase assays (Supplemental Fig. S1B–F). These validation experiments confirm that the expressions measured by CHEQ-seq are reliable and reproducible. Overall, CHEQ-seq allows cloning of hundreds of predefined enhancers into a complex barcoded library and generates reliable and reproducible reporter expression representing the functionality of a subset of these enhancers.

### Only 40% of TP53 ChIP-seq peaks are functional enhancers

**Figure 2.**
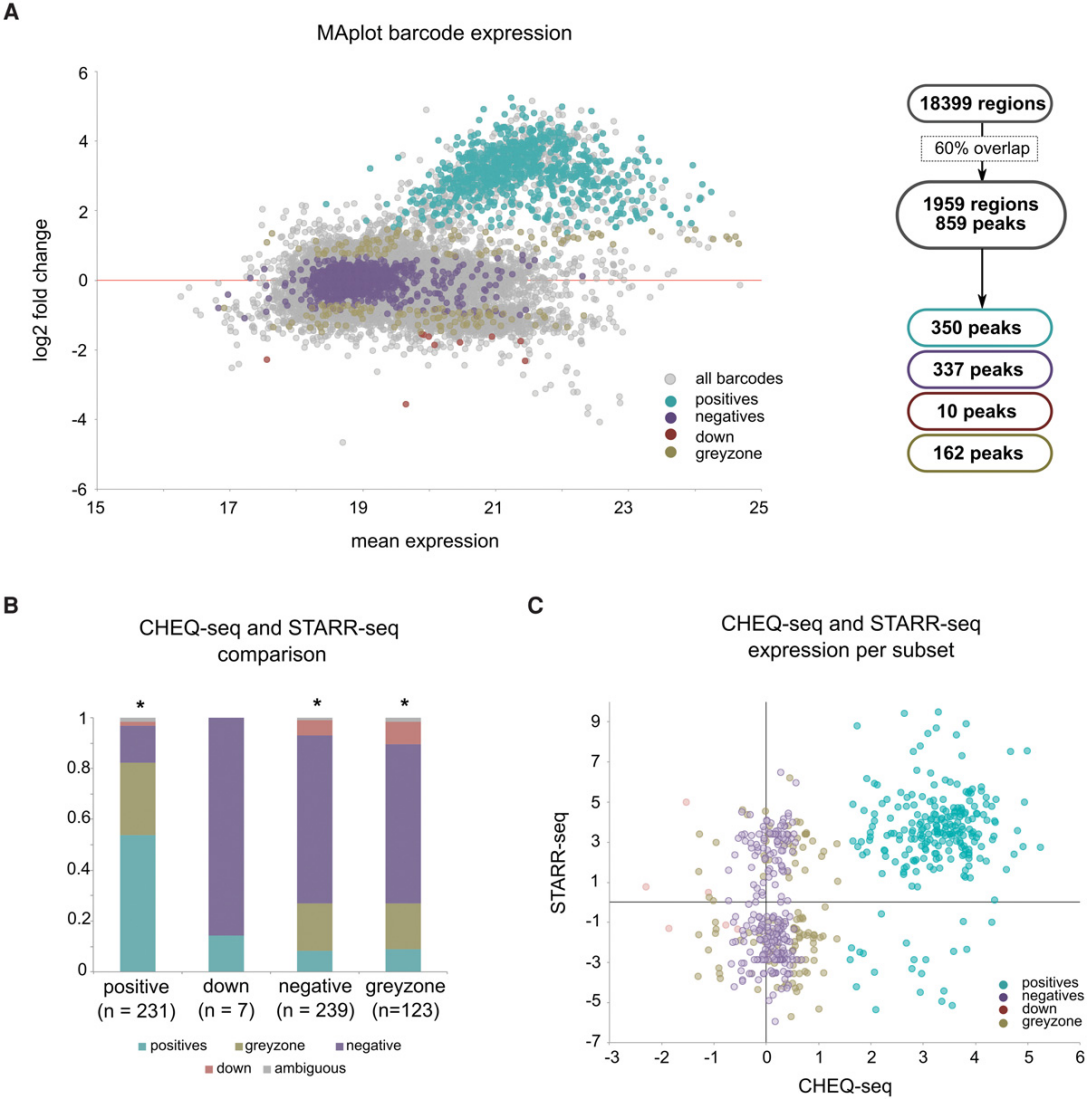
CHEQ-seq identifies TP53 responsive enhancers. (*A*) MAplot showing the distribution of CHEQ-seq barcode expression levels versus the fold induction, showing a large number of activation (green), and almost no repression (red). Negatives and grayzone are defined by thresholds on fold-change and significance (Methods). (*B*) CHEQ-seq positives are mostly positive or grayzone in STARR-seq (first bar), whereas down-regulated regions could not be confirmed by STARR-seq (second bar): (*) *P*-value <0.001 as determined by χ^2^. (*C*) Positives from CHEQ-seq are also mostly (91%) positives in STARR-seq.

Having established CHEQ-seq as an accurate multiplex enhancer-reporter assay, we turned to all the tested regions covering TP53 ChIP-seq peaks. Of the 1526 targeted peaks, 1010 (66%) were represented by at least one captured sequence and nonambiguous barcode. An additional 49 peaks were covered by randomly captured sequences. Of the 859 peaks that were covered sufficiently (Supplemental Fig. S2A; Supplemental Figs. S6, S7, 60% overlap), only 350 (40.7%) are significantly higher in p53-high compared to p53-off conditions (called “positives,” adj. *P*-value <0.05 and log2FC ≥ 1.5) (Methods). Interestingly, TP53 does not seem to directly repress enhancers since only 10 peaks show significant down-regulation upon TP53 activation (down, adj. *P*-value <0.05 and log2FC ≤ −1.5). Of the remaining ChIP-seq peaks, 337 are TP53-unresponsive (negatives), whereas another 162 show borderline expression patterns (grayzone) (Fig. 2A; Supplemental Table S2). Note that within the promoters of HKG, negative regions, or nonspecifically captured regions, almost no TP53 inducible enhancers are found (0.95%, of HKG and negative controls and 1.6% of nonspecific regions) (Supplemental Figs. S6, S8).

Next, we compared the barcode reporter activity levels measured by CHEQ-seq to the existing multiplex enhancer-reporter method STARR-seq (Arnold et al. 2013), using the same captured fragments and the same transfection conditions (Supplemental Figs. S9, S10; Supplemental Table S3). The main difference in STARR-seq is that the tested regions are inserted at the 3′ of the transcription start site, causing the enhancers to transcribe themselves rather than relying on barcodes. STARR-seq yields reproducible expression values for 975 peaks, of which 242 peaks are TP53 responsive, 463 peaks are nonresponsive, and 216 peaks are grayzone (Supplemental Fig. S11; Supplemental Table S4). The slightly smaller number of TP53 responsive elements compared to CHEQ-seq may possibly be due to differences in location of the enhancer regions within the reporter construct. STARR-seq also confirms that only very few ChIP-peaks (45 peaks) are repressed, and that the control groups do not show TP53-dependent changes (Supplemental Fig. S12). In total, 600 ChIP-seq peaks have reporter activity data from both the CHEQ-seq and STARR-seq method. When looking at the subsets created independently for each method, we see a highly significant overlap, with 190 of 231 CHEQ-seq positives being labeled as positives or grayzone in STARR-seq (82.3%, χ^2^ *P*-value = 1.24 × 10^−26^) (Fig. 2B,C). Interestingly, the small number of down-regulated ChIP-seq peaks identified by either method was not validated by the other method, suggesting that these were mainly false positives and that TP53 exclusively activates gene expression. In conclusion, multiplex enhancer-reporter assays provide reproducible sets of direct TP53 enhancers and reveal that a relatively small subset of ChIP-seq peaks act as enhancers.

### Unsophisticated TP53 enhancer logic

Using CHEQ-seq, we discriminated true positive TP53 target enhancers from unresponsive yet TP53-bound regions. As these regions are all tested in the same episomal reporter environment, the enhancer-determining information should be contained within the DNA sequence. To compare the sequences between both sets, we used three motif discovery tools, namely i-cisTarget (Imrichová et al. 2015), RSAT peak motifs (Thomas-Chollier et al. 2011), and HOMER (Heinz et al. 2010), allowing for de novo motif discovery as well as enrichment of known motifs, using libraries of position weight matrices (PWM). All tools identified the TP53 motif as most overrepresented in the TP53-responsive sequences, with highly significant *P*-values (HOMER *P*-values ≤10^−322^, RSAT significance value = 72.30, i-cisTarget NES > 28). Note that although some other motifs were found marginally overrepresented, these findings were not consistent across tools, were enriched at much lower *P*-values, and occurred in a limited number of the positive regions (maximally 25%) (Supplemental Fig. S13–S15). This suggests that TP53 mainly functions alone, without other regulatory factors cobinding at the DNA level. This is surprising as one of the proposed mechanisms for TP53 target specificity is through the recruitment of coregulatory transcription factors (Koutsodontis et al. 2001; Thornborrow and Manfredi 2001).

**Figure 3.**
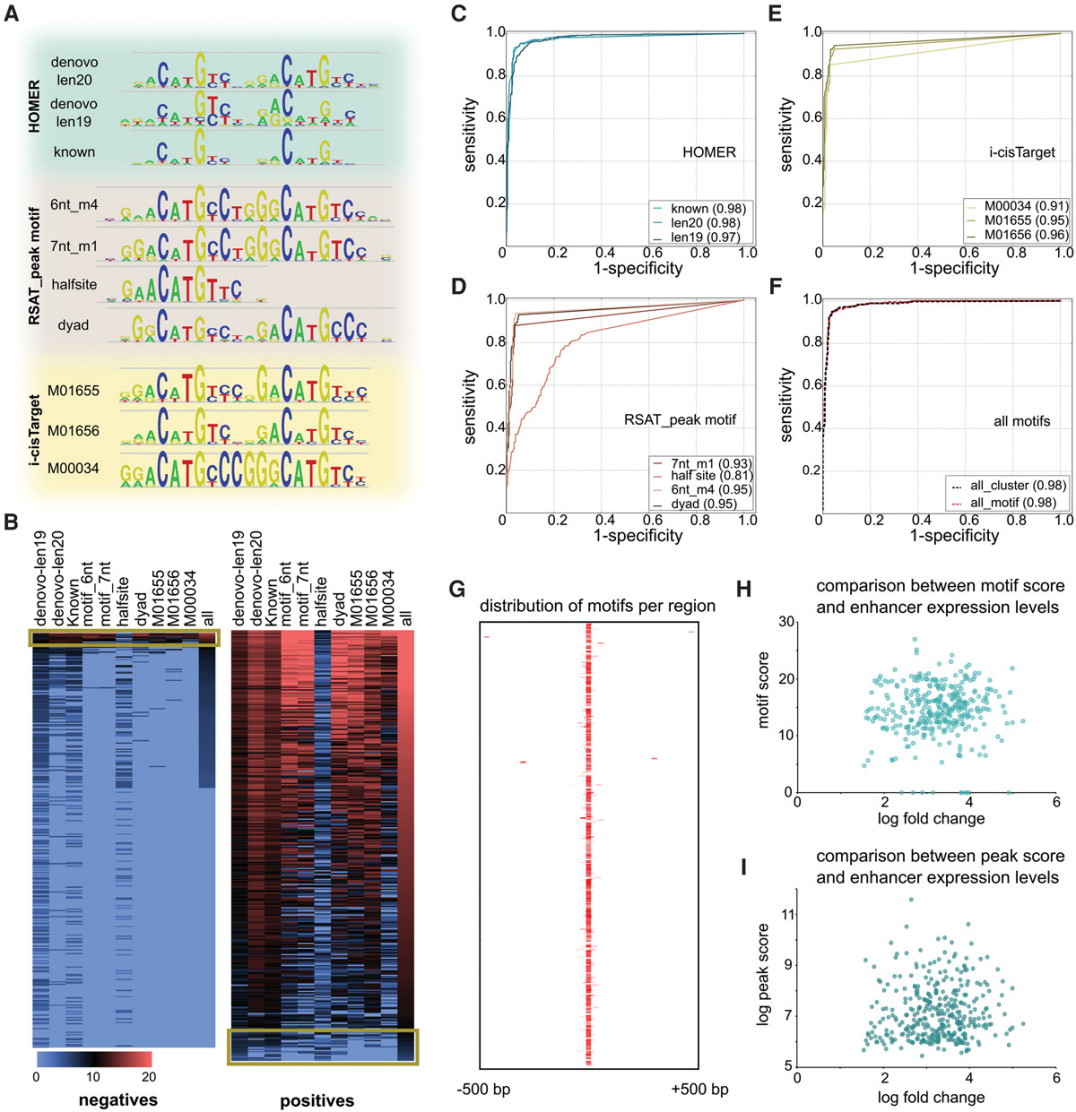
TP53 enhancer model. (*A*) Ten motifs were selected from motif discovery tools i-cisTarget, HOMER, and RSAT peak motif. (*B*) Heatmap showing the best Cluster-Buster score for each peak of the nonresponsive (negatives) and TP53-responsive (positives) subset. Discordance between PWM scores and CHEQ-seq are indicated with a yellow box. (*C*–*E*) Classification accuracy for each PWM shown as ROC curves. (*F*) Comparison of a single maximum score (red) versus a homotypic cluster of motifs (black dashed). (*G*) All positive regions with 1-kb flanking sequence centered on the best scoring motif illustrating the absence of binding site clusters. (*H*,*I*) TP53 enhancers are binary on/off enhancers as shown by the lack of correlation between the motif score and barcode expression (*H*), as well as the lack of correlation between the peak score and the barcode expression (*I*).

Previous reports indicate that small differences in the motif composition can influence binding affinity and determine target specificity (Inga et al. 2002; Wei et al. 2006; Godar et al. 2008; Smeenk et al. 2008). We therefore tested whether individual motifs, differing slightly from one another, perform differently in identifying true targets. We selected the 10 best TP53 motifs based on their significance and low occurrence in the negative set. These 10 motifs differ in length and composition, but all retain the essential double C/G core of the TP53 binding site (Fig. 3A). When plotting the maximum score of each motif for both the positive and negative sets, the TP53 motif strongly distinguishes the sets with a marked absence of motifs among the nonresponsive regions (Fig. 3B). Only 45 of 687 peaks were misclassified by CHEQ-seq, likely representing technical limitations of the experimental method itself. Indeed, the CHEQ-seq negatives that do have a TP53 motif are often identified as positive in STARR-seq (four of the six). Vice versa, CHEQ-seq positives without TP53 binding site are often not identified as positive by STARR-seq (only six of the 17). Note that almost no down-regulated enhancers identified by CHEQ-seq score for a TP53 motif (Supplemental Fig. S16).

The classification performance of these motifs, and combinations thereof, can be assessed using a Receiver Operating Characteristic (ROC) (Fig. 3C–E). Overall the performance for the different motifs is very comparable, with multiple motifs yielding an area under the ROC curve (AUC) above 0.95. A twofold cross validation at the level of feature selection (i.e., de novo motifs) ensured that these models are not overfitted (Methods; Supplemental Fig. S17). Interestingly, accurate TP53 binding site prediction requires the full PWM of the TP53 tetramer, as one half-site alone has a poor predictive performance (AUC = 0.81). In addition, and contrary to previous reports (Tokino et al. 1994; Cook et al. 1995; Riley et al. 2008), all TP53 motifs, both de novo and known, have no gap between the two half sites. As each of the individual motifs has a slightly different nucleotide composition flanking the C/G quadruple, we wondered whether scoring with a combination of all motifs would improve the predictive power (Fig. 3F). Surprisingly this was not the case as the combination of all motifs had the same predictive power as the best-scoring motif (AUC = 0.98). This suggests that the nucleotide composition of the TP53 motif can be largely captured by a single optimal PWM. Next, we tested whether homotypic clusters of TP53 sites were characteristic for TP53 responsiveness. Although previously suggested (Bourdon et al. 1997), our data suggest that a single TP53 site is sufficient to distinguish TP53-responsive from nonresponsive enhancers (Fig. 3F,G). Although the presence of a TP53 binding site is predictive of enhancer activity, the strength of the binding site is not indicative of the quantitative levels of reporter, suggesting a binary on/off state of a TP53 enhancer (Fig. 3H). This is further confirmed by comparing the quantitative enhancer-reporter levels with the height of the ChIP-seq peak score, which also does not correlate beyond the on/off categories (Fig. 3I; Supplemental Figs. S16, S18). In conclusion, TP53 enhancers are unsophisticated in their architecture with the presence of a single TP53 binding site containing a double C/G core that is both necessary and sufficient to actively drive expression in a binary fashion.

### Indirect ChIP-seq peaks have no regulatory function

**Figure 4.**
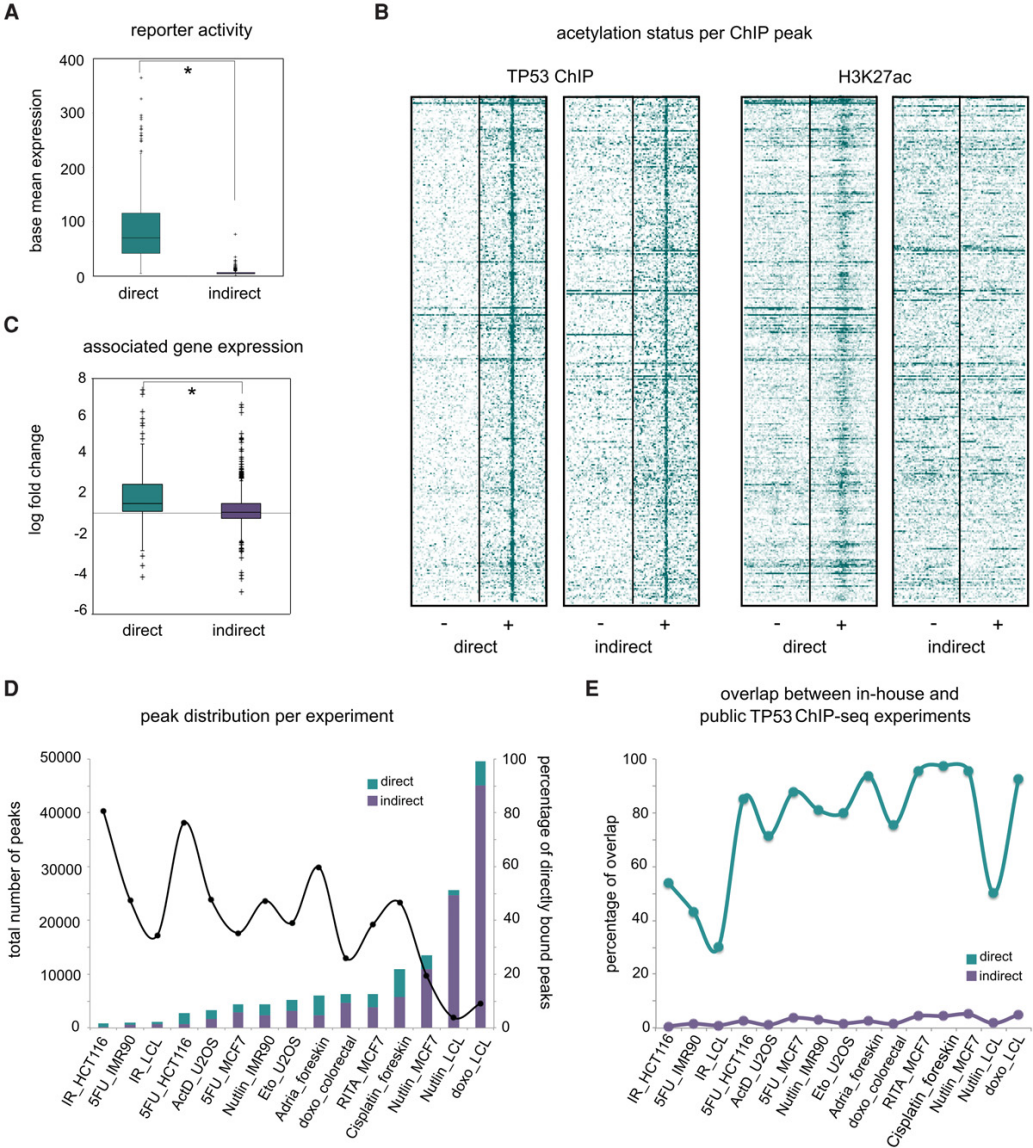
Only directly bound peaks behave as enhancers. (*A*) The average base mean expression values indicate that the direct and indirect peaks show significantly different reporter activity levels (*P*-value = 3.28 × 10^−51^). (*B*) Comparison of TP53 ChIP-seq signal and H3K27ac ChIP-seq signal between positives and negatives. Peaks are extended to 2000 bp each side. Heatmaps show the raw tag count coverage per peak. (*C*) The average differential expression of genes near (<20 kb) peaks: (*) *P*-value 4.73 × 10^−8^. (*D*) Comparison of the proportion of direct versus indirect ChIP-seq peaks across 15 publicly available TP53 ChIP-seq data sets. Experiments are ordered along the *x*-axis based on total number of peaks called. (*E*) Directly bound peaks agree, but indirectly bound do not, between in-house ChIP-seq peaks and other data sets. The percentage overlap is compared to the in-house peaks.

The ChIP-seq peaks that were not directly bound by TP53 through a TP53 binding site showed no increased enhancer-reporter activity or any basal level of enhancer activity in the reporter assay (Fig. 4A). To test whether indirect peaks may have another regulatory function, we first predicted which ChIP-seq peaks within the full set of 3634 TP53 ChIP-seq peaks (Janky et al. 2014) are likely directly bound based on the presence of a TP53 motif. To this end, we used a random forest model trained on the CHEQ-seq positive set, with the nine TP53 PWMs identified above (Supplemental Fig. S19). This classifier predicted 671 direct and 2963 indirect peaks. To investigate whether the indirect peaks could work as enhancers in their endogenous genomic context, we performed ChIP-seq against H3K27ac under the same conditions. These data confirm that only the directly bound peaks are enhancers with H3K27ac marks (Fig. 4B). This figure also shows that the direct peaks are overall higher and wider than the indirect peaks, although the distributions overlap with each other (Fig. 4B; Supplemental Fig. S20). Additionally, although the directly bound regions show increased chromatin accessibility upon TP53 activation, this is not the case for indirectly bound regions (DNase-seq data under similar conditions) (Supplemental Fig. S21; The ENCODE Project Consortium 2012; Thurman et al. 2012). Furthermore, whereas the direct peaks are located near TP53 target genes, as determined by gene annotation or by up-regulated gene expression, the indirect peaks are not enriched near putative target genes and are often found to overlap coding exons (Fig. 4C; Supplemental Figs. S22, S23; Supplemental Tables S5, S6). Finally, direct peaks significantly overlap with long terminal repeats (LTRs); indirect peaks do not (Supplemental Fig. S24).

None of the above tested features suggest that the negative indirect peaks have a regulatory function. We therefore considered the possibility that they could represent crosslinking artifacts (Waldminghaus and Skarstad 2010; Keren and Segal 2013; Baranello et al. 2015). If so, then these artifacts should not be reproducible across different experimental conditions. To test this, we turned to other publicly available data sets and collected 15 different TP53 ChIP-seq experiments performed in seven different cell lines under different TP53-stimulating conditions (Supplemental Table S7; Smeenk et al. 2011; Nikulenkov et al. 2012; Zeron-Medina et al. 2013; Botcheva and McCorkle 2014; McDade et al. 2014; Sánchez et al. 2014; Desantis et al. 2015; Hünten et al. 2015; Sammons et al. 2015). After calling peaks for each experiment, we categorized all peaks in each data set as direct or indirect using the random forest model. Whereas the total number of peaks differs greatly between data sets, this difference can be mainly attributed to differences in the number of indirect peaks (Fig. 4D). Remarkably, in contrast to the direct peaks that are strongly conserved between experiments, the indirect peaks are largely unique to each data set (Supplemental Figs. S4E, S25), strongly suggesting a nonfunctional role. In conclusion, the presence of an unsophisticated TP53 binding model is predictive of functional enhancers among ChIP peaks, whereas remaining peaks have no obvious regulatory function and may represent crosslinking artifacts.

### Genome-wide TP53 responsive enhancers are invariably used across cell types

**Figure 5.**
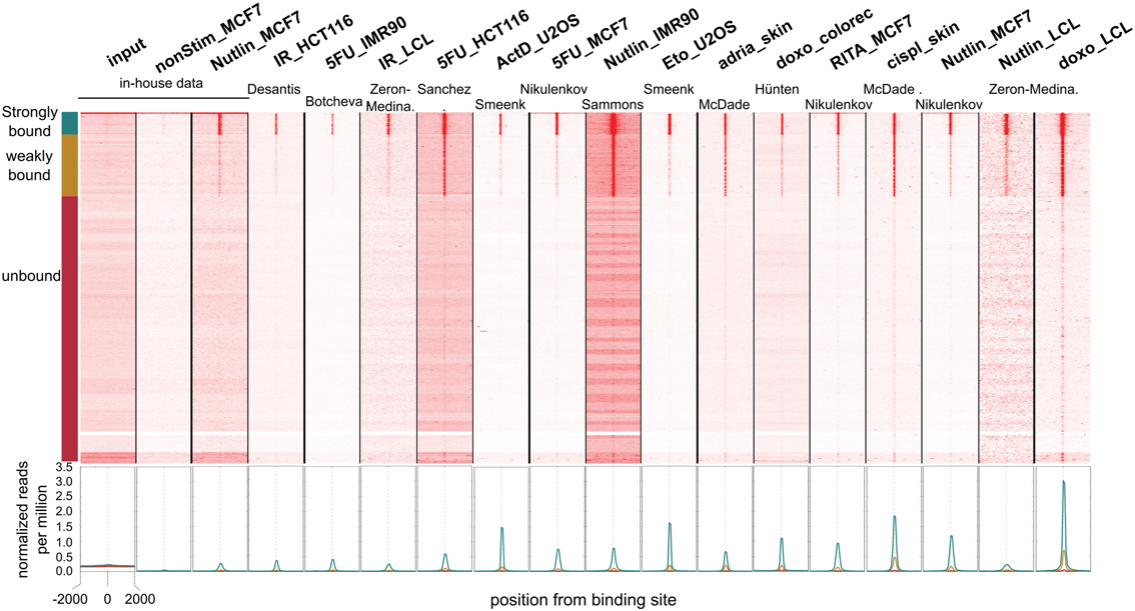
TP53 binding is conserved across data sets. Fifteen public data sets containing ChIP-seq against TP53 under various conditions were collected and remapped (Supplemental Table S7). Throughout the genome, 21,649 predicted TP53 binding sites are clustered based on their coverage across all data sets and can be subdivided into shared strong (green), shared weak (yellow), and shared unbound (red) regions. On the *bottom*, individual aggregation plots show the coverage for each cluster per sample.

Unsophisticated enhancer logic, with only a single high-scoring TP53 binding site being necessary and sufficient for a TP53 responsive enhancer, would predict invariable genomic binding across different cell types and experimental conditions. This simple model would thus contradict previous reports that proposed a direct role for TP53 binding sites and enhancers in differentially regulating cell-type–dependent activation of its targets. To address this, we decided to test our simple model on a genomic scale and across cell types and treatment conditions. We first applied our previously trained random forest model on the entire human genome and predicted 21,659 potential TP53 responsive enhancers. We then plotted the ChIP-seq signal across all publicly available data sets for all these binding sites. After testing several different clustering parameters, we found that the average coverage across binding sites within each generated cluster always converged onto three robust clusters (Methods; Supplemental Fig. S26). A first cluster (strongly bound) with 1148 sites is preferentially bound by TP53 across all data sets. A second cluster (weakly bound), with 3147 sites is also shared across data sets, but with significantly lower binding signal. Finally, a third cluster with the remaining 17,364 sites shows no binding across the data sets (Fig. 5). This finding suggests that TP53 binds only a limited number of sites throughout the genome; importantly, in contrast to earlier reports, these sites are highly conserved across different experimental conditions and cell types (Supplemental Fig. S27).

### Sequence context and DNA shape of TP53 responsive enhancers are different but not predictive

**Figure 6.**
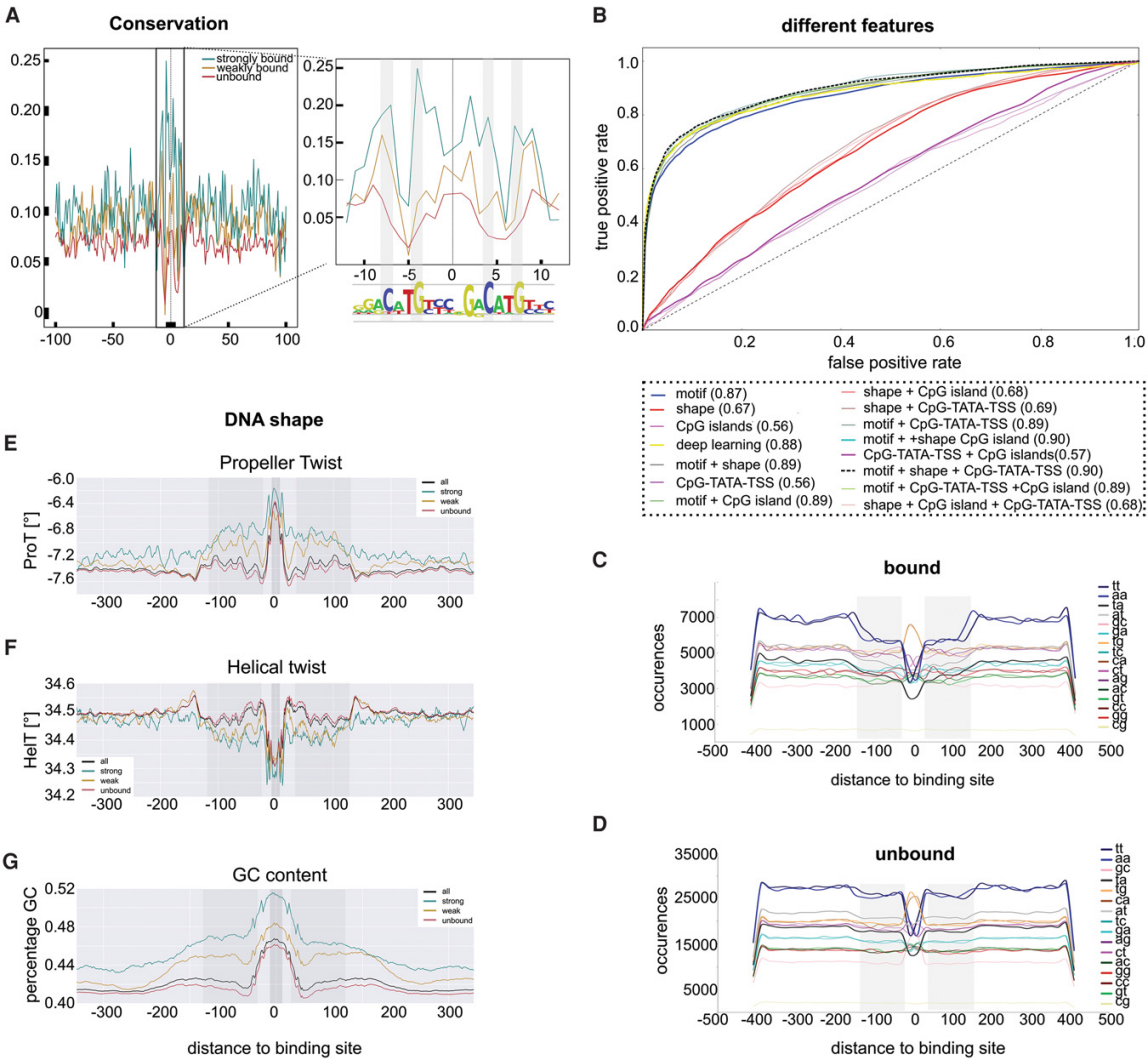
DNA features of TP53 responsive enhancers. (*A*) Sequence constraint (phastCons) of the DNA sequence around the predicted TP53 binding sites for the three classes (strongly bound, weakly bound, unbound): (*inset*) zoom in of the TP53 binding sites shows the highest conservation around the core C and G nucleotides. (*B*) Different features and different machine learning methods were tested individually and in combination for their ability to discriminate strongly bound from unbound binding sites. (*C*,*D*) Dinucleotide composition of the 800-bp sequence around the binding sites. Bound sequences (*C*) show depletion of TT and AA (blue lines) and AT and TA (gray-black lines) at 100 bp flanking the binding site compared to unbound sequences (*D*). (*E*–*G*) DNA shape features within 700 bp sequences around the predicted binding sites. Gray region ∼100 bp away from the binding site shows the strongest differences between bound and unbound sites.

To explain why TP53 preferentially binds to only a small subset (19.8%) of all the genome-wide predicted binding sites and to improve our predictive model, we investigated several local characteristics of the enhancer itself, including the sequence constraint across species, the strength of the TP53 motif, dinucleotide composition, and the DNA shape flanking the motif. Additionally, we investigated several characteristics of the genomic locus outside the enhancers, such as the presence of a nearby TATA or CpG promoter. Including these properties outside the enhancer is inspired by the possibility that binding of TP53 could perhaps be stabilized when it results in an effective target gene regulation, resulting in longer and thus higher frequency binding across cells in culture. In support of the three clusters (strong, weak, and unbound), we found that sequence constraint across vertebrate genomes is much higher for the strongly bound sites than for the weakly and unbound sites, corroborating their functional role (Fig. 6A). Note that we decided not to incorporate this feature into our predictive model because it is not a primary feature of the genome. We compared all other features, both local and global, by including them one by one and in combinations, as features into our random forest classifier. Surprisingly, only the TP53 PWMs contributed significantly to the performance. In other words, the TP53 motif not only allows distinguishing direct from indirect ChIP-seq peaks (see above), but it also further discriminates strongly bound sites from unbound sites in the genome (AUC = 0.87) (Fig. 6B, blue curve). When investigating the feature importance in the random forest model, we found the TRANSFAC motifs M01655 and M01656 to have the highest weights (Supplemental Fig. S28). The poor predictive performance of the other sequence features is surprising because on average many of these features show distinct patterns between the strongly bound and unbound sites. For example, functional TP53 sites show a drop in the occurrence of A/T dinucleotide sequences (TT, AA, TA, and AT), ∼100 bp on each side of the binding site (Fig. 6C,D). Second, the strongly bound sites differ from unbound sites in DNA shape properties such as propeller twist, helical twist, and GC content, again ∼100 bp each side of the binding site (Fig. 6E–G). Note that the weakly bound sites have an intermediate profile, having values for these features halfway between the strong and unbound site. This suggests that this cluster should indeed be considered as a separate group within the TP53 binding sites. To test whether additional unknown sequence features could play a role in determining strongly bound TP53 enhancers, we also trained a deep learning model directly on the bound versus unbound sequences, which automatically learns discriminative features (Methods). The classification performance is comparable to the random forest model using TP53 PWMs, which suggests that no additional features could be identified (Fig. 6B, yellow curve). In conclusion, a key role is played by the TP53 motif in defining functional binding, whereas other features contribute only marginally to the binding specificity.

### Strength of binding site predicts quantitative TP53 binding

**Figure 7.**
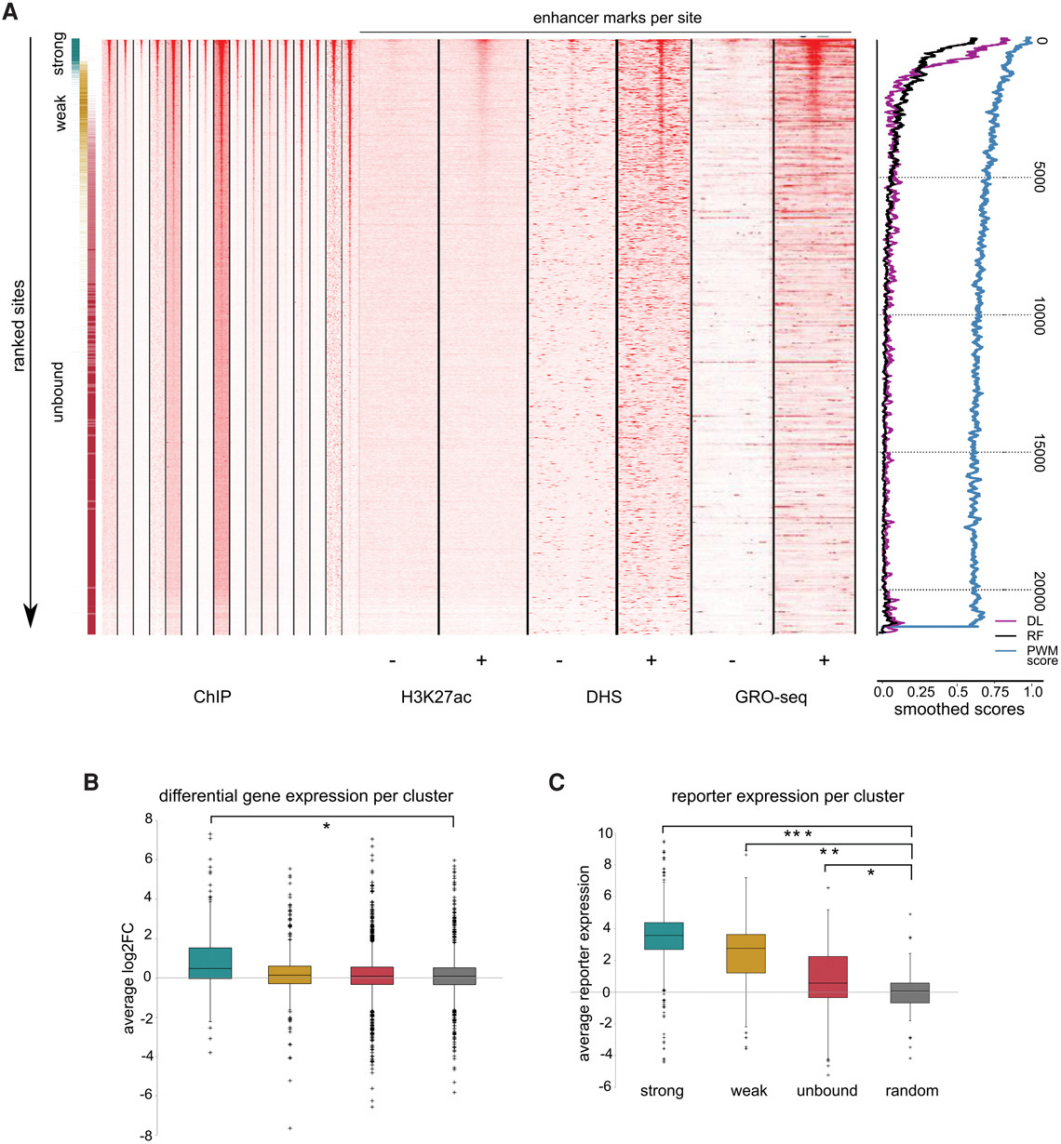
Quantitative prediction of functional TP53 sites. (*A*) Heatmap showing the TP53 ChIP-seq coverage across all 16 data sets for the approximately 21,000 predicted sites, after rank aggregation, alongside H3K27ac, DNase-seq, and GRO-seq status in untreated and TP53-stabilizing conditions. The color gradient on the *left* indicates the original clusters from Figure 5. Smoothed scores on the *right* show gradual decline with the meta-ranking: (DL) deep learning; (RF) random forest; (PWM score) position weight matrix score. (*B*) For each binding site within the clusters, the closest gene within 20 kb was assigned and the average differential expression was calculated using RNA-seq data. Only strongly bound sites associate with genes that are up-regulated upon TP53 stimulation compared to random control: (*) *P*-value = 2.45 × 10^−10^. (*C*) CHEQ-seq barcode reporter increase is also correlated with the level of binding, as compared to random regions as control: (*) *P*-value = 9.83 × 10^−49^; (**) *P*-value = 4.06 × 10^−27^; (***) *P*-value = 3.55 × 10^−5^.

Although the strongly bound and unbound clusters are clearly separated in their ability to bind TP53, the weakly bound sites exhibit intermediary characteristics. To avoid the arbitrary cutoffs of the clustering, we instead ranked all the 21,659 sites using the ChIP-seq coverage across each experiment, followed by a rank aggregation step yielding a final meta-ranking (Methods). This meta-ranking strongly recapitulates the three clusters from above (Fig. 7). Interestingly, the ChIP-seq signals are quantitatively comparable between different experiments. Furthermore, decreasing ChIP-seq signals are strongly correlated with decreasing probability of TP53 binding both by the random forest and the deep learning models (Fig. 7A, black and purple curves). The ranked list is also correlated with the functionality, as shown by correlating H3K27ac signal, DNase-seq, and GRO-seq data. This observation provides additional confirmation that the regions are ranked in decreasing functionality (Fig. 7A). Note that many of the weakly bound sites also show increased chromatin accessibility, which is expected when TP53 binds and displaces nucleosomes, but they have significantly less H3K27ac and GRO-seq marks (Sammons et al. 2015; Su et al. 2015). This functional difference is furthermore corroborated by the fact that genes located near highly ranked sites are located near TP53-related genes, whereas lower ranked sites show no Gene Ontology or pathway enrichment for TP53-related processes (Supplemental Tables S8, S9). Nevertheless, weakly bound sites can function as enhancers, since 49% of weakly bound sites are positive in the CHEQ-seq or STARR-seq assay, compared to nearly 80% of the strong sites driving gene expression in the context of a reporter assay (Fig. 7C; Supplemental Figs. S29, S30). Taken together, these results suggest that functionality is predictable by the sequence and decreases gradually by the strength of the TP53 binding site.

## Discussion

The functional annotation of all regulatory elements in the noncoding part of the human genome is a key challenge in genome biology. Although biochemical events such as protein binding to the DNA occur very frequently, and such events cover >80% of the genome (The ENCODE Project Consortium 2012), only a fraction of these binding events is expected to be functional (Carvunis et al. 2015). In the context of the presented work, we consider a genomic region to be functional if it contributes in a deterministic and observable fashion to the regulation of gene expression. In our study, we found that among the 95,077 observed binding events for TP53, across sixteen data sets covering diverse cell types and conditions, and among the more than 20,000 possible TP53 recognition sites in the genome, only a small fraction are bona fide TP53 responsive enhancers. Rather than deciding on a cutoff, we generated a ranking of all candidate sites based on the combination of the aggregated binding data and found that this meta-ranking correlates strongly with the strength of the TP53 binding site. This correlation can already be observed with a high-quality position weight matrix (PWM), but is even stronger for a trained classifier based on multiple PWMs or a deep learning model trained directly on strongly bound enhancer sequences. We provide a hub at the UCSC Genome Browser, containing genome-wide binding across experiments and the scores for the model predictions (Methods). This TP53 hub can serve as a reference for TP53-related future studies. Indeed, we argue that the observed consistency of direct TP53 binding across experimental conditions is so high that our ranking can be a good guide for the putative functionality of a given TP53 site, regardless of the experimental conditions. Note that the models presented here are derived from data sets after inducing TP53 in cell culture, both in cancer and normal cell lines. The question whether this unsophisticated enhancer model for TP53 also applies to other TP53-related functions, for example during in vivo development, remains to be investigated.

Only a small, yet predictable, subset of experimentally determined binding events represents TP53 responsive elements. The other binding events (up to >90%, depending on the data set) (Fig. 4D,E) are presumably the result of cross-linking artifacts or other technical aspects of the ChIP method or its analysis. Similar observations have been made before, in which binding of a transcription factor to the DNA can occur either due to the presence of its motif, when the binding is functional, or independently of the motif, when the binding is not functional. For example, Kvon et al. (2012) found that Twist is often bound to HOT regions in *Drosophila*, but only the binding in the mesoderm at the right time point, when Twist is actually expressed, corresponded to motif-dependent direct binding sites.

In this study, we have learned several new things about TP53. First, our data support the most common model for TP53 binding, namely that TP53 binds the DNA strictly as a tetramer, to a duplicate of the consensus palindromic responsive element RRRCWWGYYY (R = purine; W = adenine/thymine; Y = pyrimidine), separated by a spacer of length N = 0, and not to single half-sites. Whereas previous reports argued that the sequence composition of the TP53 binding site may play a role in explaining context-dependent activation (Szak et al. 2001; Weinberg et al. 2005; Beckerman and Prives 2010), our results are consistent with other studies that reject this hypothesis (Wei et al. 2006; Smeenk et al. 2008). Another clear result from our experiments is that TP53 can only directly activate enhancers and in contrast to previous reports (Hoffman et al. 2002; Godar et al. 2008), we find no evidence of direct repression. Another intriguing finding is that TP53 seems to bind to chromatin independently of preexisting nucleosome accessibility. For this, we confirm earlier studies (Lidor Nili et al. 2010; Cui et al. 2011; Su et al. 2015). This finding supports the clutch-like model, whereby transcription factors are able to displace nucleosomes, and are in competition with nucleosome binding. Finally, we find that TP53 mostly acts alone as a TF bound to its target enhancers, although previous studies had suggested cofactorship at the DNA level (Koutsodontis et al. 2001; Thornborrow and Manfredi 2001). Note that non-sequence-specific cofactors (e.g., EP300) are likely to interact with TP53 at the protein level, independent of the DNA sequence, to recruit RNA polymerase and activate target gene transcription.

Enhancer sequences have recently been shown to share characteristic features beyond transcription factor binding sites, such as a particular distribution of Cs, Gs, and CpGs (Kwasnieski et al. 2012) or the presence of dinucleotide repeat motifs, such as CA, GA, or CG (Yáñez-Cuna et al. 2014). The flanking nucleotides of our TP53 responsive elements also show an intriguing bias in nucleotide composition, which likely represents preferential nucleosome binding positions. Although these patterns are clearly visible at the global level, these features do not have predictive power.

Among the approximately 21,000 potential TP53 binding sites, we find about 1000 regions that are strongly bound and represent bona fide responsive elements, regulating target gene expression, as effectors of the TP53 response. In addition, another subset of around 3000 sites show weak TP53 binding. This set is much less related to enhancer activity, gene expression, or nearby gene function. On the other hand, the recognition sites are more conserved in evolution, and they have stronger PWM matches than the unbound. It is intriguing to speculate what the function of these sites could be. One possibility is that these sites could be preferentially bound when there is an excess of TP53 protein. Indeed, from a statistical point of view, given that TFs need “time” to find their functional binding sites, a cell needs to produce an excess of functional transcription factors to ensure that the entire functional *cistrome* remains occupied. Thus, at all times, a fraction of molecules is undergoing fast binding turnover at nonfunctional sites (also called treadmilling) (Lickwar et al. 2012), in which they become fixated during the ChIP protocol. Interestingly, we find a quantitative relationship between prolonged residence and the strength of the TP53 binding site, rather than other additional sequence features. This idea adheres to the recently proposed clutch-like model of transcription factor binding (Lickwar et al. 2012).

Achieving high-confidence predictions at the genome-wide scale requires machine learning classifiers that are trained on large sets of positive and negative enhancer sequences. We, and others before us, have shown that massively parallel enhancer-reporter assays can relatively quickly lead to such training sets, and usually lead to exciting new insight into the *cis*-regulatory logic of enhancers (Kwasnieski et al. 2012; Melnikov et al. 2012; Arnold et al. 2013; Kheradpour et al. 2013; White et al. 2013). In our study, we tested long enhancer sequences, of several hundreds of base pairs. Earlier methods for massively parallel enhancer-reporter assays (MPRA) often relied on oligonucleotide synthesis to generate sequences, thereby limiting the fragment size considerably below that of an average metazoan enhancer (∼200 bp as compared to ∼500–800 bp) (LeProust et al. 2010). This issue has recently been overcome by cloning captured fragments into a barcoded reporter assay (Shen et al. 2015). In addition, the STARR-seq method bypasses the issue of short input fragments by inserting randomly fragmented regions of the genome straight into a library downstream from a reporter gene rather than upstream (Arnold et al. 2013). STARR-seq was originally developed for *Drosophila*, in which the genome size is manageable to clone in its entirety. However, considering that the human genome is 25 times larger, it presents a considerable challenge toward genome-wide assays. A solution to this is to preselect the input, as has been done in the preset study, as well as recently in mice, using a STARR-seq-like approach, called CapStarr-seq (Vanhille et al. 2015). The possibilities that these methodologies provide with regard to enhancer validation, both in vitro and in vivo (White et al. 2013; Shen et al. 2015), underscore the value and the need for such approaches in the field of regulatory genomics today.

Our case study of TP53 may seem peculiar in the sense that TP53 acts in isolation, without coregulatory transcription factors that bind to the same enhancer. Although this was quite an unexpected finding, in retrospect this observation fits well within the cell-autonomous function of TP53. Indeed, upon DNA damage, TP53 activates the appropriate target genes to either repair the damage or launch the apoptotic program (Beckerman and Prives 2010). Although we now have a better understanding of the TP53 *cistrome*, it remains to be discovered how generic activation of the same set of enhancers is differentially steered in the currently operational gene regulatory network, and how the cellular context further contributes to the resulting responses like apoptosis or growth arrest.

The unsophisticated enhancer model, with only a single TP53 binding site, is to our knowledge the first report of such a new class of enhancers in the human genome. Previously, such enhancer models were used for compact genomes from bacteria, or sometimes yeast, but not for multicellular organisms. Plant and metazoan enhancers are usually classified using the enhanceosome or billboard model (Arnosti and Kulkarni 2005), but these types of models are based on the combinatorial action of multiple transcription factors to reach specificity. Whereas developmental enhancers need to integrate multiple signals and environmental queues (e.g., signaling gradients), each cell can be considered independently responsible for its own genome integrity. Such a model may be valid for additional cell-autonomous factors, such as the cell cycle–related factors of the E2F family. Thus, we consider the TP53 enhancer model to represent a new class of unsophisticated, single-factor metazoan enhancers.

## Methods

### CHEQ-seq plasmid and bait design

A super core promoter, a synthetic intron (Arnold et al. 2013), and a venus reporter gene (Roure et al. 2007) were inserted between the KpnI and XbaI restriction sites of the pGL4.23 plasmid (Promega Catalog No. E8411). Barcodes were incorporated using an inverse PCR into the modified pGL4.23 backbone with AscI (see Supplemental Material). One hundred twenty nanograms of plasmid was electroporated per 20 µL of electrocompetent cells (Invitrogen, Catalog No. C6400-03) and extracted using a Giga prep (Qiagen No. 12191). ChIP-seq peaks for TP53 were called against input (GSE47043) as described before (Janky et al. 2014) and were filtered for centromere and telomere regions and ranked based on their peak score. The top 1700 regions were selected for bait design as described in Supplemental Materials and Methods.

### Generating the CHEQ-seq and STARR-seq libraries

Genomic DNA was extracted, adapter ligated, and amplified. Targeted regions were captured with the MYbaits protocol (Custom bait libraries, MYcroarray). At least three captures were performed and pooled after purification. The CHEQ-seq plasmid containing the barcode pool was linearized and combined with ∼250 ng input DNA in a total of four infusion reactions (Clontech). The recombined library was precipitated overnight and transformed at 100 ng per 20 µL electrocompetent cells. See Supplemental Materials and Methods for additional details.

### Cell work and extractions

MCF7 cells (*TP53* wild type or *TP53* knockdown) were cultured and transfected as described before (Janky et al. 2014). DNA and RNA were extracted and cDNA prepared according to the manufacturer's guidelines. See Supplemental Materials and Methods for more details.

### Library preparations

cDNA or (extracted) plasmid DNA was amplified with two rounds of PCR using the Phusion High-fidelity PCR master mix (Catalog No. M0532S; PCR details in Supplemental Materials and Methods). The library was purified using the AMPure XP Beads (Beckman Coulter, Catalog No. A63880) and sequenced on the Illumina HiSeq2500 platform. For STARR-seq, the DNA and cDNA libraries were created as described before (Arnold et al. 2013). For PacBio sequencing, the input library was amplified using the Phusion High-fidelity PCR master mix (Catalog No.M0532S; PCR details in Supplemental Materials and Methods). The linear fragments of ∼2–3 kb were purified using AMPure XP Beads (Beckman Coulter, Catalog No. A63880). Libraries were subsequently sent for SMRT PacBio sequencing (Pacific Biosciences).

Data processing and subset determination for both CHEQ-seq and STARR-seq libraries are described in Supplemental Materials and Methods.

### Motif discovery and motif scoring

HOMER (Heinz et al. 2010) and RSAT peak motifs (Thomas-Chollier et al. 2011) were run on the positive set with the negative set as background. i-cisTarget (Imrichová et al. 2015) was run on the positive set. For HOMER, length of the motif was set at length 19 or 20 (-len) to allow for discovery of the consensus TP53 motif in the de novo option. Motifs were selected based on their overall performance and low occurrence in the negative set (Supplemental Materials and Methods). For each obtained motif or the combination of all 10 motifs, Cluster-Buster (Frith et al. 2003) was used to score the positive and negative sets using –c 0 and –m 0. The highest motif score (or CRM score) for each region was obtained and used to determine the predictive value of each motif to classify regions into positives or negatives. In short, the sensitivity and specificity for each motif was calculated, and the area under the receiver operating characteristic curve (AUC) determined.

### ChIP-seq, RNA-seq, and public data

ChIP-seq against H3K27ac was performed and analyzed as described before (Verfaillie et al. 2015). RNA-seq for MCF7 *TP53* knockdown was extracted and performed as described previously (Janky et al. 2014). The collected public ChIP-seq data against TP53 are summarized in Supplemental Table S7. See Supplemental Materials and Methods for a more extended methodology and processing of the data.

### Random forest model and feature-vector representation

Different random forest models were generated (Supplemental Materials and Methods). As random forest implementation, we used the scikit-learn Python package. Each classifier uses an ensemble of 151 decision trees. The parameter max_features (responsible for number of features to consider when looking for the best split) was set to sqrt (number of features). To calculate the feature importance, we used the Gini impurity criterion averaged across trees, using the whole training data. The quality of each model was estimated in fivefold cross validations. For each PWM, the motif score was calculated using a Hidden Markov Model as implemented in Cluster-Buster (Frith et al. 2003). Number of coding genes and lncRNAs was calculated using BEDTools (Quinlan and Hall 2010) and a custom bash script. The file with TSSs of genes was downloaded from the UCSC Genome Browser. High confident subsets of lncRNAs were downloaded from LNCipedia (Volders et al. 2015). Files with the positions of promoters with TATA-box and/or GpC islands have been downloaded from the FANTOM5 resource (Lizio et al. 2015).

### seqMINER, clustering, and DNA shape

BAM files of all public data (15 samples) and in-house data (see GSE47043) were loaded into seqMINER (Ye et al. 2011). A BED file with all predicted TP53 binding sites was loaded. Alternatively, in-house TP53 ChIP-seq data, H3K27ac, or DNase-seq BAM files were loaded and compared across the positive and negative CHEQ-seq peaks. The flanking area was set at 2000 bp around the binding site. Heatmaps show the raw tag count coverage from each BAM file for each input site or peak. Determination of clustering is described in Supplemental Materials and Methods and in Supplemental Figure S26. DNA shape data indicating Helix Twist (HelT) and Propellor Twist (ProT) for HG19 were downloaded from ftp://rohslab.usc.edu/hg19/ in bigWig format (Chiu et al. 2015) and analyzed as described in Supplemental Materials and Methods.

### Prediction of TP53 binding using deep learning

The network was trained using the RMSprop algorithm for Stochastic Gradient Descent with 100 training samples in each minibatch and binary cross-entropy loss function for minimization. The Keras 0.2.0 library (https://github.com/fchollet/keras) with the Theano 0.7.1 backend was used for implementation. Calculations have been performed with NVIDIA K40c accelerator. The regularization parameters are dropout proportion (fraction of outputs randomly set to 0) for layer 2: 10%; layer 3: 10%; layer 6: 50%; all other layers: 0%. The details of the CNN model architecture are listed in Supplemental Materials and Methods.

## Data access

Data generated for this study have been submitted to the NCBI Gene Expression Omnibus (GEO, https://www.ncbi.nlm.nih.gov/geo/) under accession number GSE76657. The predicted genome-wide TP53 binding sites and combined ChIP-seq data from all experiments used in this work are available as a track hub (http://ucsctracks.aertslab.org/p53/hub.txt). To activate this track hub, go to My Data, Track hubs in the UCSC Genome Browser menu, and provide this URL in “My Hubs.”
